# Retinoids Regulate Adipogenesis Involving the TGFβ/SMAD and Wnt/β-Catenin Pathways in Human Bone Marrow Mesenchymal Stem Cells

**DOI:** 10.3390/ijms18040842

**Published:** 2017-04-15

**Authors:** Jun Cao, Yuhong Ma, Weiqi Yao, Xiaoye Zhang, Dongcheng Wu

**Affiliations:** Department of Biochemistry and Molecular Biology, School of Basic Medical Sciences, Wuhan University, Wuhan 430071, China; Juncao@whu.edu.cn (J.C.); yuhongma@whu.edu.cn (Y.M.); weiqiyao@whu.edu.cn (W.Y.); xiaoyezhang@whu.edu.cn (X.Z.)

**Keywords:** human bone marrow mesenchymal stem cell, adipogenesis, retinoid, retinoic acid receptor β (RARβ), TGFβ/SMAD pathway, Wnt/β-catenin pathway

## Abstract

Retinoids may regulate cell differentiation as ligands of retinoic acid receptors (RARs) and/or retinoid X receptors (RXRs). We showed that RAR agonists promoted adipogenesis by upregulating the expression of CCAAT/enhancer-binding protein β (C/EBPβ) in the early stages, but blocked adipogenesis at a later stage in human bone marrow mesenchymal stem cells (hBMSCs). RXR agonists promoted adipogenesis at all time points in hBMSCs. The effect of RAR agonists was mediated mainly by the RARβ subtype. RAR agonists, in contrast to RXR agonists, significantly promoted the expression of RARβ. Knockdown of the RARβ gene via small hairpin RNA (shRNA) attenuated the inhibition of RAR agonists toward adipogenesis. Furthermore, we found that RAR agonists upregulated the transforming growth factor β (TGFβ)/SMAD pathway and Wnt/β-catenin pathway on adipogenesis in hBMSCs, and the stimulating effects were noticeably decreased with the RARβ gene knockdown. Both RAR agonists and RXR agonists inhibited adipogenesis and blocked the promoter activity of C/EBPβ and peroxisome proliferator-activated receptor γ (PPARγ) in SW872 cell. These results indicated the RAR agonists perform dual roles in adipogenesis in hBMSCs, and the TGFβ/SMAD pathway and Wnt/β-catenin pathway may involve the inhibitory effect of RAR agonists. RARβ is the main receptor subtype mediating the effect. The roles of RXR agonists in adipogenesis exhibited cell type-specific differences, and may be based on the integration of signals from different RXR dimers.

## 1. Introduction 

Bone marrow mesenchymal stem cells (BMSCs) show a self-renewal ability and may have multi-lineage differentiation potential, as they can differentiate into adipocytes, osteoblasts, and chondroblasts [1].

Adipogenesis requires the sequential activation of numerous pro-adipogenic transcription factors, including CCAAT/enhancer-binding proteins (C/EBPs) and peroxisome proliferator-activated receptor γ (PPARγ) [2]. C/EBPs regulate the expression of adipocyte-specific genes and promote adipocyte differentiation. C/EBPβ and C/EBPδ are expressed during the very early stages of adipogenesis and their expression decreases in the terminal stages. C/EBPβ, in concert with C/EBPδ, induces the expression of C/EBPα and PPARγ, which control the late stages of adipogenesis. There are C/EBP-binding sites in the promoter for PPARγ [3]. PPARγ is a master regulator of adipogenesis. PPARγ is activated by ligands, such as thiazolidinedione (TZD), and PPARγ/retinoid X receptor (RXR) heterodimers regulate the transcription of adipocyte target genes. The liganded PPARγ/RXR heterodimers bind to the peroxisome proliferator response elements (PPREs) in target genes (e.g., *FABP4*) and modulate the activity of the basal transcription machinery [4].

Retinoids, metabolites, or analogs of vitamin A function as ligands for retinoic acid receptors (RARs) and/or RXRs. RARs include three isotypes, RARα, RARβ, and RARγ, and act as gene transcription regulators through heterodimerization with RXRs (RXRα, β, and γ) [5]. Natural, endogenous retinoids include all-trans retinoic acid (ATRA), which bind to, and activate, RARs, and 9-cis retinoic acid (9CRA), which binds to, and activates, both RARs and RXRs [6]. The RXR/RAR heterodimer can bind to RAREs (retinoic acid DNA response elements) in the promoter regions of target genes. RXRs also form RXR/RXR homodimers, which bind target DNA sequences called RXREs (retinoid X response elements). Several retinoids (e.g., SR11237) can induce RXR homodimer binding to RXREs [6]. RXRs are also heterodimeric partners of several other nuclear receptors, including the PPARs, liver X receptor (LXR) and vitamin D receptor (VDR), and these heterodimers are categorized as either permissive or non-permissive. Heterodimers with PPARγ are considered permissive, and ligands of RXR can activate the functions of PPARγ/RXR with non-liganded PPARγ [7].

Retinoids regulate adipogenesis and lipid metabolism, which are mediated by RAR/RXR and RXR/RXR. RARs are negative regulators of osteoblast and adipocyte differentiation [8]. Retinoic acid (RA) inhibits growth at a high concentration (100 μM) and inhibits differentiation at a low concentration (0.1–10 μM) in preadipocytes [9]. RA downregulates the expression of adipogenic transcription factors PPARγ and C/EBPα in 3T3-L1 adipocytes [10]. RA can activate the cellular retinoic acid binding protein 2 (CRABP-II)/RARγ pathway by upregulating the expression of the adipogenesis inhibitors preadipocyte factor 1 (Pref-1), SRY (sex determining region Y)-box 9 (Sox9), and Kruppel like factor 2 (KLF2) to block adipogenesis and suppress diet-induced obesity [11]. RXR homodimers may promote adipogenesis by activating the target genes of PPARγ [12]. The RXR ligand LGD1069 increases the expression of adipogenesis-related genes, including *FABP4*, *adipsin*, and *PPARγ*, in mammary carcinoma [13,14].

Transforming growth factor β (TGFβ)/SMAD signaling pathway is involved in the regulation of cell differentiation. TGFβ promotes osteogenic differentiation while, at the same time, inhibiting adipogenic differentiation under adipogenic differentiation conditions in hMSCs [15]. TGFβ inhibits adipogenic differentiation in human preadipocytes, which is mediated mainly by SMADs [16,17]. SMAD3 inhibits adipogenic conversion, whereas interfering with SMAD3 function confers resistance to inhibition of adipogenesis by TGFβ [18].

The Wnt signaling pathway plays important roles in adipogenic differentiation. Wnt10b activates canonical Wnt signaling to downregulate the expression of C/EBPα and PPARγ and inhibits adipogenesis in preadipocytes [19]. Knockdown of β-catenin may induce spontaneous adipocytogenesis [20]. Canonical Wnt3a increases β-catenin levels and induces dedifferentiation of both 3T3-L1 and human adipocytes [21]. Inhibition of Wnt4 or Wnt5a expression blocks fat accumulation and downregulates the expression of adipogenic genes [22]. Wnt5b, together with Wnt5a, stimulates the expression of PPARγ and FABP4, induces adipocyte differentiation and inhibits β-catenin-dependent Wnt signaling at the initiation of adipogenesis [23].

Here, we report that retinoids regulated adipogenic differentiation in human bone marrow mesenchymal stem cells (hBMSCs) via RARβ, involoving the activation of the TGFβ/SMAD and the Wnt/β-catenin signaling pathway.

## 2. Results

### 2.1. Retinoids Affected Proliferation and Adipogenic Differentiation in a Concentration- and Time-Dependent Manner in hBMSCs

**Figure 1:**
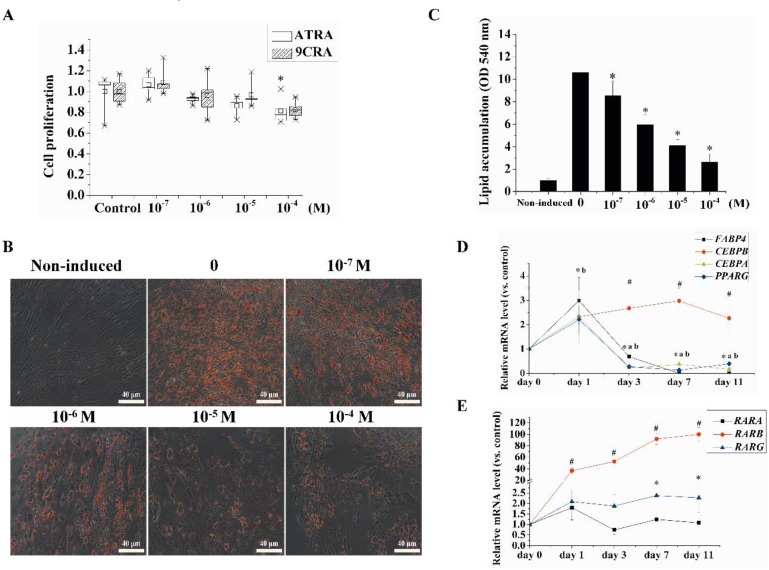
Effects of different concentrations of retinoids on proliferation and adipogenic differentiation of human bone marrow mesenchymal stem cells (hBMSCs). (**A**) Cells were treated with different concentrations of all-trans retinoic acid (ATRA) and and 9-cis retinoic acid (9CRA) for three days, the cell proliferation was assessed using methylthiazolyldiphenyl-tetrazolium bromide (MTT) assay. * (ATRA), *p* < 0.05, versus control. The assay was repeated three times independently; (**B**,**C**) Cells were treated for seven days with the indicated concentrations of ATRA, and the lipid accumulation was determined by Oil Red O staining. The images were captured at low magnification (20×). Oil Red O was quantified at an absorbance of 540 nm; (**D**,**E**) Cells were treated with 10 μM ATRA for seven days, and quantitative reverse transcription polymerase chain reaction (qRT-PCR) was used to determine the mRNA levels of adipogenic genes and retinoic acid receptors (RARs). Gene expression was analyzed using fold-change comparisons to the corresponding control group at the same time point. Gene expression levels were normalized to the internal reference gene *ACTB*. (**A**) # (*CEBPB*), * (*PPARG*), a (*CEBPA*), b (*FABP4*), *p* < 0.05, versus the day 0. (**B**) # (*RARB*), * (*RARG*), *p* < 0.05 versus day 0.

To determine the effect of retinoids on cell proliferation, hBMSCs were treated with different concentrations of ATRA (0.1–100 μM) or 9CRA (0.1–100 μM) for three days, Cell viability was assessed using the methylthiazolyldiphenyl-tetrazolium bromide (MTT) assay. As shown in Figure 1A, ATRA had a statistically significant (*p* < 0.05) effect on inhibiting the proliferation of hBMSCs at 100 μM after three days of treatment; however, there was no significant difference at ATRA (0.1–10 μM) and 9CRA (0.1–100 μM).

To investigate the effect of retinoids on adipogenic differentiation in hBMSCs, we cultured primary hBMSCs and induced adipogenesis for seven days with the application of adipogenic differentiation medium containing different concentrations of ATRA. The accumulation of lipids was assessed by Oil Red O staining and quantified by spectrophotometry. ATRA (above 0.1 μM) significantly inhibited adipogenic differentiation seven days after ATRA was added, and this inhibition was concentration-dependent (Figure 1B,C).

We also used quantitative reverse transcription polymerase chain reaction (qRT-PCR) to investigate the effects of ATRA (10 μM) on the expression of adipogenic genes and RARs subtypes during adipogenesis using qRT-PCR. Expression levels of the adipogenic genes *PPARG* and *FABP4* were significantly upregulated 24 h after the addition of ATRA, and expression of *CEBPB* was significantly upregulated after three days and was maintained at a high level until 11 days. However, levels of *CEBPA*, *PPARG*, and *FABP4* began to significantly decrease after three days. Among RAR subtypes, *RARB* significantly increased after 24 h and reached a 100-fold increase on day 11. The expression levels of the other subtypes, *RARA* and *RARG*, showed only minor increases (Figure 1D,E).

### 2.2. The Adipogenic Differentiation of hBMSCs Was Inhibited by RAR Agonists and Promoted by RXR Agonists

**Figure 2:**
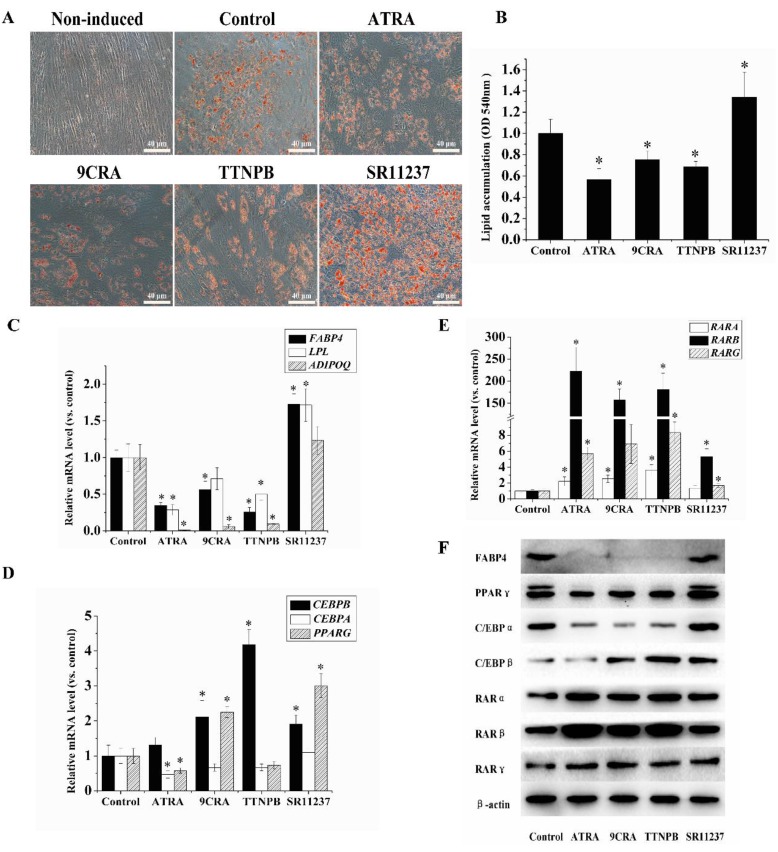
The effects of different types of retinoids on hBMSCs adipogenic differentiation. (**A**,**B**) During adipogenic differentiation, cells were treated with 10 μM ATRA, 9CRA, 4-[(1E)-2-(5,5,8,8- tetramethyl-5,6,7,8-tetrahydro-2-naphthalenyl)-1-propen-1-yl] benzoic acid (TTNPB), or SR11237 for seven days. Lipid accumulation was determined by Oil Red O staining. The images were captured at low magnification (20×). Oil Red O was quantified at an absorbance of 540 nm; (**C**–**F**) qRT-PCR and Western blotting were used to determine the mRNA and protein levels of adipogenic genes and RARs. β-Actin was used as an internal control. * *p* < 0.05, compared with control. PPARγ: peroxisome proliferator-activated receptor γ; C/EBP: CCAAT/enhancer-binding protein; FABP4: fatty acid binding protein 4.

To investigate the effects of RAR and RXR agonists on adipogenic differentiation in hBMSCs, hBMSCs were placed in adipogenic differentiation medium with an RAR agonist ATRA or 4-[(1E)-2-(5,5,8,8-tetramethyl-5,6,7,8-tetrahydro-2-naphthalenyl)-1-propen-1-yl] benzoic acid (TTNPB) or an RXR agonist (9CRA or SR11237) for seven days, with each agonist used at 10 μM. Oil Red O staining was used for the determination of lipid accumulation (Figure 2A,B). Adipogenic differentiation was attenuated by 10 μM ATRA, 9CRA, and TTNPB on day seven compared to the control group. In contrast, 10 μM SR11237 promoted cell differentiation. 

We next examined the effects of RAR and RXR agonists on the expression of adipogenic genes and RARs using qRT-PCR and Western blotting. As shown in Figure 2C–F, 10 μM ATRA, 9CRA, and TTNPB decreased the expression levels of *FABP4, LPL, ADIPOQ, CEBPA*, and *PPARG*. Conversely, 10 μM 9CRA and TTNPB increased the expression of *CEBPB* at seven days after their addition. Ten micromoles of SR11237 significantly promoted the expression *FABP4, LPL, CEBPB*, and *PPARG* on day 7. Furthermore, ATRA, 9CRA, or TTNPB at 10 μM promoted the expression of RARs (*RARA, RARB,* and *RARG*). Among these RARs, the expression of *RARB* was the most pronounced (with a fold change of 222.864 in the ATRA treatment group compared to the control). SR11237 elicited the smallest increase in the expression of the RARs (with a fold change of 5.303 for *RARB* compared to the control).

### 2.3. Effect of Retinoids on the Expression of TGFβ/SMAD Pathway-Related Genes in hBMSCs

**Figure 3:**
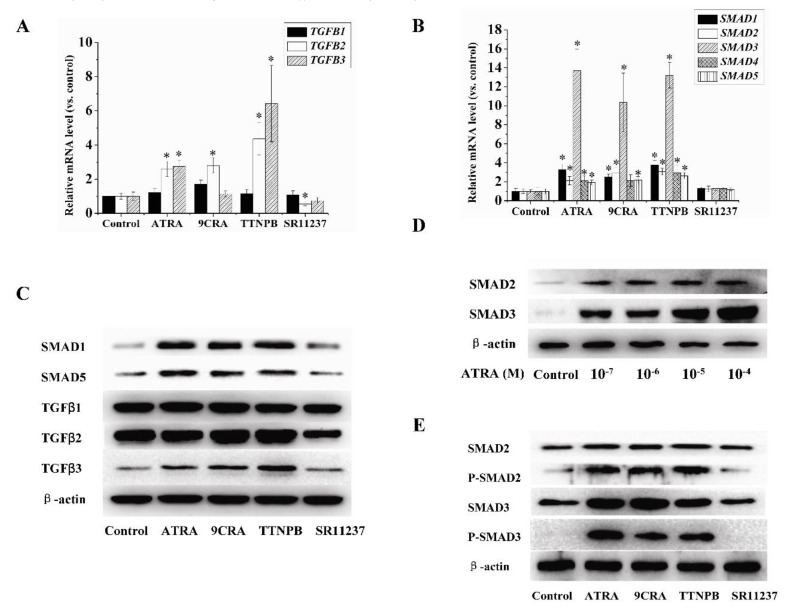
The effects of RAR and RXR agonists on the expression of genes related to the transforming growth factor β (TGFβ)/SMAD pathway. (**A**–**E**) human bone marrow mesenchymal stem cells (hBMSCs) were induced to become adipocytes by using adipogenic differentiation medium with or without 10 μM ATRA, 9CRA, TTNPB, or SR11237 for seven days. qRT-PCR and Western blotting were used to detect the mRNA and protein levels of TGFβ/SMAD pathway genes. β-actin was used as an internal control. * *p* < 0.05 versus the control group.

TGFβ/SMAD pathway is a key pathway related to adipogenesis [18]. To understand the mechanism driving the effects of RAR and RXR agonists on adipogenic differentiation in hBMSCs, we investigated the role of the TGFβ/SMAD pathway in this process. qRT-PCR showed that 10 μM ATRA, 9CRA, and TTNPB promoted the expression of SMAD1-5 and TGFβ2-3 ( Figure 3A,B). The change in SMAD3 expression was the most notable, which was consistent with findings from a previous study [24]. Moreover, 10 μM SR11237 did not demonstrate a stimulatory effect on SMAD3, but did show a slight inhibition of TGFβ2. Western blot analysis showed that 10 μM ATRA, 9CRA, or TTNPB promoted the protein levels of SMAD1–5 and TGFβ2–3. Ten micromoles of ATRA increased the protein levels of SMAD2 and SMAD3 in a concentration-dependent manner. Furthermore, 10 μM ATRA, 9CRA, or TTNPB promoted phosphorylation of SMAD2 and SMAD3. Additionally, 10 μM SR11237 decreased the protein levels of TGFβ2, but had no effect on other tested proteins belonging to the TGFβ/SMAD pathway (Figure 3C–E).

### 2.4. Effect of Retinoids on the Expression of Wnt Pathway-Related Genes in hBMSCs

**Figure 4:**
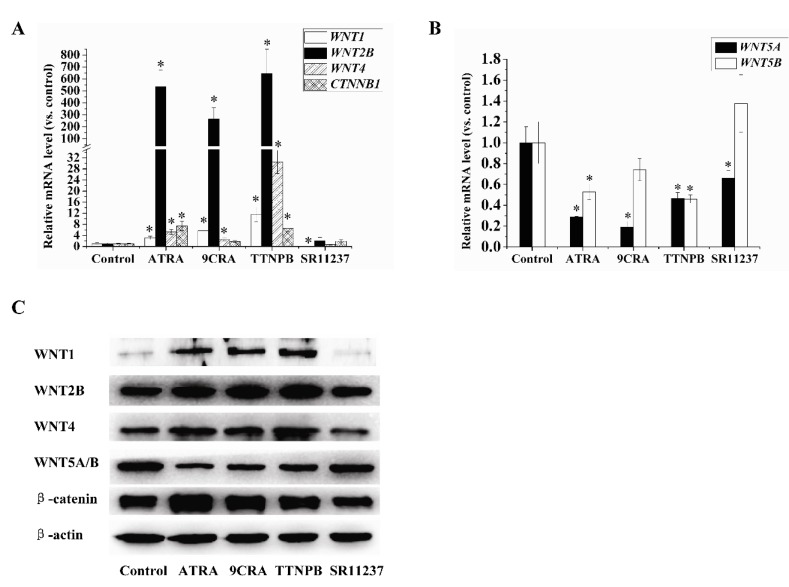
The effects of RAR and RXR agonists on the expression of genes related to the Wnt pathway. (**A**–**C**) hBMSCs were treated with 10 μM ATRA, 9CRA, TTNPB, and SR11237 for seven days during adipogenic differentiation. qRT-PCR and Western blotting were used to detect the mRNAs and protein levels of genes related to the Wnt pathway. β-actin was used as an internal control. All data are shown as means of three independent experiments ± standard deviation. * *p* < 0.05, versus control group.

The Wnt pathway is involved in the regulation of adipogenic differentiation. We investigated the effects of retinoids on the expression of genes related to the Wnt pathway. As shown in Figure 4A–C, 10 μM ATRA, 9CRA, and TTNPB increased the mRNA and protein levels of *WNT1, WNT2B, WNT4*, and *CTNNB1*, in which the upregulation of *WNT2B* expression was the most notable, but decreased the expression levels of *WNT5A* and *5B*. In contrast, 10 μM SR11237 decreased the mRNA and protein levels of *WNT1*. SR11237 also decreased the mRNA levels of *WNT5A*, but not as obviously as the RAR agonists. Protein levels of WNT4, WNT2B, WNT5A/B, and β-catenin in the SR11237 treatment group did not change significantly compared with the control group. *WNT2* and *WNT3A* expression levels were not detected by qRT-PCR and Western blotting.

### 2.5. Knockdown of the RARB Gene Attenuated the Effect of Retinoids on Adipogenic Differentiation in hBMSCs 

**Figure 5:**
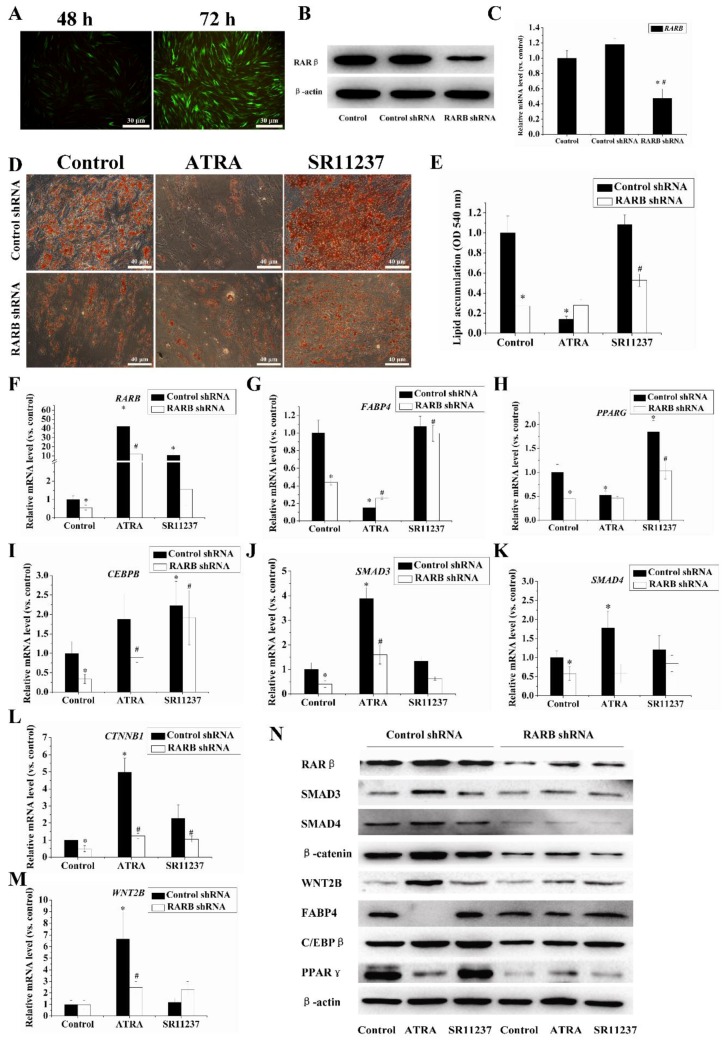
The knockdown of *RARB* by shRNA attenuated the effects of retinoids on adipogenesis. (**A**) Cells were treated with copGFP control lentiviral particles. The images were captured at low magnification (20×) with a fluorescence microscope (Olympus IX73, Olympus, Tokyo, Japan) at 48 h and 72 h; (**B**,**C**) The efficiency of RARβ shRNA lentiviral particles in hBMSCs was measured by qRT-PCR and Western blotting. β-actin was used as an internal control. * *p* < 0.05, compared with the control group. # *p* < 0.05, compared with the control shRNA treatment group. Data are representative of three independent experiments; (**D**,**E**) Cells from the RARβ shRNA lentiviral particles treatment group and the control shRNA group were induced by adipogenic differentiation medium with or without 10 μM ATRA or SR11237 for seven days. Lipid accumulation was determined by Oil Red O staining. The images were captured at low magnification (20×). Oil Red O was quantified at an absorbance of 540 nm; (**F**–**N**) The gene expression levels of *RARB*, *CEBPB*, *PPARG*, *FABP4*, *SMAD3*, *SMAD4*, *WNT2B*, and *CTNNB1* were assessed by qRT-PCR and Western blotting. β-actin was used as an internal control. * *p* < 0.05 compared with the control group of the control shRNA treatment cells, # *p* < 0.05 compared with the control group of the RARβ shRNA treatment cells.

To investigate the role of RARβ in adipogenesis, we knocked down the expression of the *RARB* gene by using RARβ shRNA lentiviral particles. qRT-PCR and Western blotting analyses showed that the RARβ level was significantly reduced in the RARβ shRNA treatment group compared to the control group (Figure 5B,C). Oil Red O staining demonstrated that seven days after the induction of adipogenic differentiation, the lipid accumulation was significantly reduced in the RARβ shRNA group than in the control shRNA group. Conversely, ATRA inhibition of adipogenesis was markedly attenuated, and SR11237 maintained a stimulatory effect in the *RARB* knockdown group (Figure 5D,E). We further measured the influence of the *RARB* knockdown on adipogenic genes, specifically SMAD3, SMAD4, WNT2B, and β-catenin using qRT-PCR and Western blotting. As shown in Figure 5E–N, levels of RARβ and adipogenic genes (*CEBPB*, *PPARG*, and *FABP4*) decreased in the *RARB* knockdown cells compared to the control cells. Meanwhile, the suppression of ATRA in adipogenesis was attenuated significantly in the *RARB* knockdown cells. Furthermore, the expression of SMAD3, SMAD4, and β-catenin decreased with the *RARB* knockdown, similar to the changes observed in the RARβ. The stimulatory effect of ATRA on the expression of SMAD3, SMAD4, WNT2B, and β-catenin was noticeably decreased.

### 2.6. Both RAR Agonists and RXR Agonists Inhibited Adipogenic Differentiation and Blocked the Promoter Activity of C/EBPβ and PPARγ in SW872 Cells 

**Figure 6:**
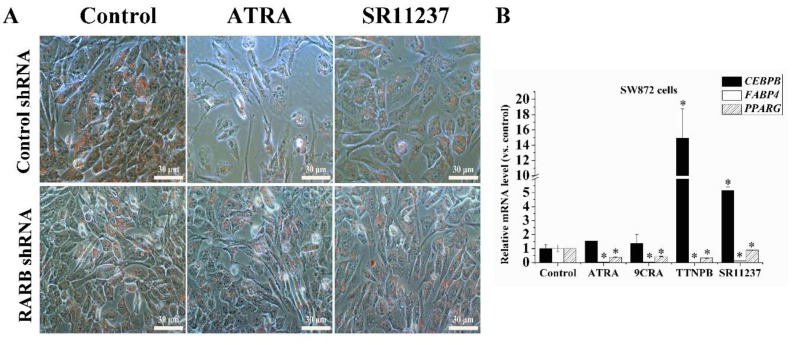

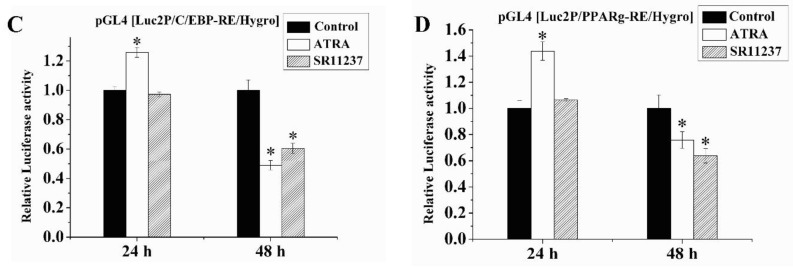
Effects of RAR agonists and RXR agonists during adipogenic differentiation in SW872 cells. (**A**) During adipogenic differentiation, cells from the RARβ shRNA lentiviral particles treatment group and the control shRNA group were treated with ATRA and SR11237 at 10 μM for seven days. Lipid accumulation was determined by Oil Red O staining. The images were captured at low magnification (20×); (**B**) qRT-PCR was used to determine the mRNA levels of adipogenic genes, and *ACTB* was used as an internal control. All data are shown as means of three independent experiments ± standard deviation. * *p* < 0.05, versus control group; (**C**,**D**) SW872 cells were co-transfected with pGL4 (luc2P/C/EBP-RE/Hygro) or pGL4 (Luc2P/PPARg-RE/Hygro) and pTK-Renilla-Luciferase plasmids. Twenty-four hours later, the cells were induced in adipogenic differentiation medium with ATRA or SR11237 at 10 μM for 24 h or 48 h. Luciferase reporter activity was normalized to Renilla luciferase activity. *p < 0.05, compared with control.

In order to further investigate the role of retinoids in different cells, we examined the effects of RAR agonists and RXR agonists in adipogenic differentiation in SW872 cells. Oil Red O staining showed that adipogenic differentiation was inhibited by 10 μM ATRA and SR11237 on day 7 compared to the control group (Figure 6A). qRT-PCR data showed RAR agonists (10 μM ATRA, 9CRA, and TTNPB) decreased the expression of *FABP4* significantly at day 7 in SW872 cells in the same way as in hBMSCs. RXR agonist (10 μM SR11237) decreased the expression levels of *FABP4* in SW872 cells, showing an opposite effect compared with hBMSCs. RAR agonists and RXR agonists decreased the expression of *PPARG* in SW872 cell while, on the contrary, 9CRA and SR11237 increased the expression of *PPARG* in hBMSCs. In addition, TTNPB and SR11237 increased the expression of *CEBPB* significantly in SW872 cells, which was consistent with the effect in hBMSCs (Figure 6B). We knocked down expression of the *RARB* gene by using RARβ shRNA lentiviral particles in SW872 cells, Oil Red O staining showed the inhibition of adipogenesis by ATRA and SR11237 was markedly attenuated in the *RARB* knockdown group, which is basically consistent with the results in hBMSCs (Figure 6A).

We examined the influences of ATRA and SR11237 in the promoter activity of C/EBPβ and PPARγ in SW872 cells using a Luciferase reporter assay. As shown in Figure 6C,D, when cells were treated with 10 μM ATRA, promoter activity of C/EBPβ and PPARγ increased at 24 h, but was significantly decreased at 48 h; treatment with 10 μM SR11237, promoter activity of C/EBPβ and PPARγ were at normal levels at 24 h, but significantly inhibited at 48 h.

## 3. Discussion

In this study, we explored the effects of retinoids on adipogenic differentiation in hBMSCs and SW872 cells. Numerous studies have shown that retinoids regulate adipogenesis. RA blocks the expression of PPARγ and C/EBPα through RARs, rather than RXRs [25]. ATRA also stimulates adipogenesis though RARs [26,27]. Our results suggest that the activation of two types of retinoic acid receptors, RARs and RXRs, show different effects in hBMSCs. The RAR agonists (ATRA and TTNPB) promoted adipogenesis in the early stages by upregulating the expression of PPARγ and FABP4 over 24 h, but they inhibited adipogenic differentiation on day 3 in hBMSCs. In contrast, SR11237, an agonist of RXRs, promoted adipogenesis at all time points in hBMSCs. Interestingly, the RXR ligand 9CRA inhibits adipogenesis. These findings are consistent with previous reports [28]. We believe that 9CRA-induced downregulation of PPARγ after three days is dependent on RARs. Like ATRA, 9CRA can bind to RARs and activate RAR/RXR heterodimers [6]. Similarly, the RXR agonist LGD1069 can bind to RARs, mostly in the form of RAR/RXR heterodimers, and inhibit adipogenesis in ST13 preadipocytes [14]. Another RXR agonist, SR11237, can bind to RXRs, but not RARs, to activate RAR/RXR heterodimers dependent of RAR ligand binding because RARs are non-permissive receptors. SR11237 can also activate RXR/RXR homodimers or PPARγ/RXR heterodimers and promote adipogenic differentiation without the PPARγ ligand [12]. 

Furthermore, we found RAR agonists also inhibited the adipogenic differentiation in SW872 cells. However, RXR agonists showed an opposite effect in adipogenic differentiation between hBMSCs and SW872 cells. SR11237 significantly inhibited the expression *FABP4* and *PPARG* on day 7 in SW872 cells. We hypothesize that the possible reason is involved in the relative intracellular levels of partners of RXRs (e.g., RARs, PPARγ) in different cell lines. If the RARs binding by endogenous retinoids have relative dominance in cells, SR11237 mainly activates the RAR/RXR heterodimers as RXR’s ligand, presenting a similar action to RAR agonists. When PPARγ is relatively dominant in cells, SR11237 may stimulate the adipogenic differentiation by activation of PPARγ/RXR or RXR/RXR dimers. The final effects of SR11237 depended on the integration of signals from different RXR dimers. We found that the effects of ATRA and SR11237 were significantly decreased with *RARB* knockdown in SW872 cells, suggesting the inhibitory effects of SR11237 were also based on the RAR/RXR heterodimers.

It is known that RA does not block C/EBPβ expression, but liganded RAR specifically represses the transcriptional activation of C/EBPβ/α in 3T3-L1 cells, and 10 μM RA inhibits C/EBPβ phosphorylation at Thr188 in 3T3-F442A cells [29,30,31]. RA also induces the nuclear enrichment of MEK1, which physically sequesters PPARγ from its target genes [32]. In the current study, we found that the RAR agonists inhibited the expression of adipogenic marker genes C/EBPα, PPARγ, and FABP4 during adipogenic differentiation at seven days in hBMSCs and SW872 cells, but the expression of C/EBPβ was increased. This is likely because C/EBPα and PPARγ are the downstream target genes of C/EBPβ, suggesting that the inhibition of adipogenic differentiation caused by RAR agonists may be related to a decrease in C/EBPβ transcription activity. Luciferase promoter activity assay showed that promoter activity of C/EBPβ and PPARγ was increased at 24 h with ATRA treatment in SW872 cells, but the activity decreased at 48 h. The results indicated the inhibitive effects of RAR agonists was partially based on the block of promoter activity of C/EBPβ and PPARγ. On the other hand, C/EBPβ and PPARγ activity did not change significantly at 24 h with RXR agonist SR11237 treatment, but also decreased at 48 h in the SW872 cell line similar to ATRA. This was consistent with the inhibitive effect of SR11237 in SW872 cells.

In this study, we found that RAR agonists significantly upregulated the expression of RARβ in hBMSCs, whereas the expression of RARα and RARγ were only slightly increased. Thus, we postulated that the inhibition of RAR agonists on hBMSCs was mainly mediated by RARβ. To verify this hypothesis, we used lentivirus-mediated shRNA to knock down RARβ expression. Our data showed that the inhibitory effects of RAR agonists were significantly decreased on day 7, that the expression of FABP4 was upregulated in the shRNA group, and that the expression of PPARγ was completely restored to the level observed in the control group. These results demonstrate that RA inhibited adipogenic differentiation of hBMSCs predominantly through RARβ. Although the inhibition induced by ATRA was mediated mainly by RARβ, the lipid accumulation was significantly reduced in RARβ shRNA cells than in control cells. These data suggest the RARβ may play a role in promoting adipogenesis. It has been reported that RARβ induce adipogenesis in mouse embryonic stem cells. The induction is dependent on the activity of glycogen synthase kinase (GSK) 3, which is a critical negative regulator of Wnt [33].

We then found that RAR agonists (ATRA, 9CRA, TTNPB), but not RXR agonists (SR11237), significantly upregulated the mRNA and protein levels of SMAD1, SMAD2, SMAD3, and SMAD5, among which SMAD3 levels increased most significantly on day 7. RAR agonists promoted the phosphorylation of SMAD2 and SMAD3. SR11237 slightly inhibited the expression of TGFβ2. When RARβ was knocked down, expression levels of SMAD3 and 4 were significantly decreased, and the stimulatory effects of ATRA on SMAD3 and 4 were noticeably attenuated. Our findings indicated that the inhibition of RARβ in adipogenesis may be mediated mainly by the TGFβ/SMAD pathway in hBMSCs. RXR agonists have no remarkable effects on the expression of TGFβ/SMAD signaling. It has been acknowledged that SMAD3 and SMAD4 can physically interact with C/EBPβ and C/EBPδ to repress the transcriptional activity of C/EBPs [34,35]. SMAD3 can interact with C/EBPβ via its Mad-homology 1 (MH1) domain and interfere with the DNA binding of C/EBPβ, RA stimulates the expression of SMAD3 and cannot inhibit adipogenesis in the absence of SMAD3 [24]. In addition, the overexpression of SMAD2 inhibits differentiation, but to a milder extent than SMAD3 [17]. Together with previous reports, our results indicated that RAR agonists most likely inhibited the promoter activity of C/EBPβ and PPARγ via the TGFβ/SMAD pathway in hBMSCs and SW872 cells.

Retinoids regulate the Wnt signaling pathway. RA increases the expression of proteins of the noncanonical Wnt pathway, including Wnt5a, 7a, and T-cell factor 3 (TCF3) , but inhibits the canonical pathway in embryonic stem cells [36]. RA treatment increases the expression and transcriptional activity of β-catenin protein, and stimulates the expression of *Wnt1* and *Wnt4* genes directly by occupancy of their promoter in 3T3-L1 cells [37]. ATRA inhibits bone morphogenetic protein 9 (BMP9)-induced adipogenic differentiation in preadipocyte through TGFβ/SMAD and Wnt/β-catenin signaling [38]. ATRA stimulates the gene expression of Wnt proteins and receptors, whereas RARγ interacts with β-catenin and inhibits the transcriptional activity of Wnt/β-catenin signaling in mouse epiphyseal chondrocytes [39]. In the present study, we found the RAR agonists (ATRA, 9CRA, TTNPB) significantly increased the expression of WNT1, WNT2B, WNT4, and β-catenin. In contrast, RXR agonist SR11237 decreased the mRNA level of WNT1. RAR agonists inhibited the expression WNT5A and WNT5B. The stimulatory effects of ATRA on WNT2B and β-catenin were noticeably attenuated with the *RARB* knockdown. These results suggested that RAR agonists promote the expression of Wnt/β-catenin by RARβ/RXR heterodimers, involving the inhibition of adipogenic differentiation. 

There is a crosstalk between Wnt/βcatenin and the TGFβ signaling pathway. TGFβ/SMAD3 upregulate the expression of Wnt2b, Wnt4, Wnt5a, Wnt9a, and Wnt11 in vascular smooth muscle cells [40], TGFβ1 induces the expression of Wnt3 and β-catenin in BMSCs and stimulates Wnt4 expression in fibroblasts [41,42]. Wnt inhibits Smad4 phosphorylation by GSK3 and potentiates the TGFβ signal [43]. In addition, the two pathways are closely related in adipogenesis. TGFβ inhibits adipogenesis in hMSCs with the Wnt signal synergistically. TGFβ1 increases the accumulation of β-catenin during adipocyte differentiation in the 3T3-L1 adipocyte. TGFβ1 inhibits expression and activity PPARγ through the β-catenin pathway [44,45,46]. Therefore, we hypothesized that the upregulation of genes related to the Wnt/β-catenin pathway are induced by the TGFβ/SMAD signaling pathway with retinoid treatment, then the Wnt/β-catenin pathway conversely promotes the activity of the TGFβ/SMAD signal, forming a positive feedback loop to drive the inhibition of adipogenesis. This will be followed up in a future study.

## 4. Materials and Methods 

### 4.1. Cell Cultures and Adipogenic Differentiation 

hBMSCs (Cyagen Biosciences, Guanzhou, China) and human liposarcoma SW872 cell line (ATCC, Rockville, MD, USA) were cultured in modified Eagle’s medium (MEM) containing 10% (*v*/*v*) fetal bovine serum (FBS), 100 units/mL penicillin, 0.1 mg/mL streptomycin, and 2 mM l-glutamine at 37 °C under a humidified, 5% CO_2_ atmosphere. Culture medium was changed every 2–3 days. When the cells were 100% confluent, they were induced to differentiate into adipocytes with adipogenic differentiation medium containing Human Mesenchymal Stem Cell Adipogenic Differentiation Basal Medium A, Mesenchymal Stem Cell-Qualified Fetal Bovine Serum (10%), glutamine (2 mM), penicillin (100 units /mL), streptomycin (0.1 mg/mL), insulin (10μg/mL), 3-isobutyl-1-methylxanthine (IBMX) (500 μM), rosiglitazone (0.5 μM), and dexamethasone (1 μM) (Cyagen Biosciences, Guanzhou, China). ATRA, 9CRA, 4-[(1E)-2-(5,5,8,8-tetramethyl-5,6,7,8- tetrahydro-2-naphthalenyl)-1-propen-1-yl] benzoic acid (TTNPB), and SR11237 (Sigma-Aldrich, St. Louis, MO, USA) was dissolved in dimethyl sulfoxide (DMSO) for the experiments. The cells were cultured under dim light when treated with retinoids to prevent their degradation.

### 4.2. Cell Proliferation Assays

Cells were seeded into 96-well plates at an initial density of 5000 cells/well and grown to 70% confluence. Cells were exposed to ATRA (0–100 μM) or 9CRA (0–100 μM) in complete medium for three days. Two-hundred microliters of MTT solution (0.5 mg/mL) was added to each well and incubated for 4 h at 37 °C. The formazan crystals were dissolved in 200 μL DMSO. The optical density (OD) that formazan formed was measured at 492 nm. Each concentration was performed in triplicate.

### 4.3. Oil Red O Staining 

In six-well plates, the cells were washed with phosphate buffered saline (PBS), fixed in 4% formalin in PBS for 30 min, and then washed once with PBS. Cells were stained with 60% Oil Red O stock solution (0.5 g of Oil Red O in 100 mL of isopropanol) for 30 min. The cells were then washed with PBS and photographed using phase-contrast microscopy. The Oil Red O was quantified by extraction in isopropanol. The OD was measured at 540 nm. 

### 4.4. Quantitative RT- PCR 

Total RNA was extracted from hBMSCs using TRIzol reagent (Invitrogen, Carlsbad, CA, USA). After quantification, total RNA was reverse transcribed into cDNA with the Goscript reverse transcription system (Promega, Madison, WI, USA) in a total volume of 20 μL according to the manufacturer’s instructions. The cDNA was assayed in a 20 μL real-time PCR reaction using SYBR Premix Ex Taq (Tli RNaseH Plus) (Takara, Dalian, China) with an ABI PRISM 7500/7500 Fast Real-Time PCR System according to the manufacturer’s instructions. The following cycle parameters were used: initial denaturation step at 95 °C for 30 s, followed by 40 cycles of 95 °C for 5 s and 60 °C for 34 s. The data were analyzed using the delta–delta *C*t method for relative quantification. All measurements represent a minimum of three independent experiments. The primer sequences were as follows (Table 1):

### 4.5. Immunoblot Analysis 

The cell total protein was collected with cell lysis buffer for Western and IP (Beyotime, Beijing, China). Protein concentration was determined using a BCA kit (Beyotime, China). The protein samples were mixed with loading buffer and heated at 100 °C for 5 min. Next, the samples were loaded in a 10% Tris-glycine sodium dodecyl sulfate (SDS)-polyacrylamide gel for electrophoresis at 90 V for 30 min in a stacking gel, and at 130 V for 60 min in a separating gel. Proteins were then transferred to polyvinylidene difluoride (PVDF) membranes at 400 mV for 90 min at 4 °C. The membranes were blocked in 5% nonfat milk for 1 h at room temperature. The primary antibodies used were anti-β-actin (1:1000, ZSGB-BIO, Beijing, China), anti-FABP4 (1:1000, Abcam, Cambridge, UK), anti-FABP4 (1:1000, Abcam), anti-PPARγ (1:1000, CST, Danvers, MA, USA), anti-C/EBPβ (1:1000, CST), anti-C/EBPα (1:1000, CST), anti-RARα (1:1000, Abcam), anti-RARβ (1:1000, Abcam), anti-RARγ (1:1000, Abcam), anti-SMAD1 (1:1000, CST), anti-SMAD2 (1:1000, CST), anti-SMAD3 (1:1000, CST), anti-SMAD4 (1:1000, CST), anti-SMAD5 (1:1000, CST), anti-TGFβ1 (1:1000, R&D), anti-TGFβ2 (1:1000, R&D Systems, Minneapolis, MN, USA), anti-TGFβ3 (1:1000, R&D), anti-Wnt1 (1:1000, Santa Cruz Biotechnology, Santa Cruz, CA, USA), anti-Wnt2b (1:1000, Abcam), anti-Wnt4 (1:1000, Abcam), anti-Wnt5a/b (1:1000, CST), and anti-β-catenin (1:1000, CST). The primary antibodies were added to 5% nonfat milk and incubated with the membranes at 4 °C overnight. The next day, membranes were washed three times with 1 × TBST (5 min per wash). The membranes were then incubated with horseradish peroxidase (HRP) conjugated goat anti-rabbit IgG (1:1000, CST) or goat anti-mouse IgG (1:5000, ZSGB-BIO, China) for 1 h at room temperature. Finally, the membranes were washed in TBST three times (5 min per wash), and the antibodies were detected using enhanced chemiluminescence (ECL) (Pro-Light HRP kit, Tiangen, Beijing, China). All experiments were performed at least three times.

### 4.6. Luciferase Reporter Assay 

The SW872 cell line at 80% confluency in a 24-well plate was transiently transfected with: pGL4 (Luc2P/C/EBP-RE/Hygro) plasmid containing a C/EBPbeta response element (Promega), pGL4 (Luc2P/PPARg-RE/Hygro) plasmid containing a PPAR response element (PPRE) (Promega) and pTK-Renilla-Luciferase plasmid (Promega) using lipofectamine 2000 transfection reagents (Invitrogen) according to manufacturer’s protocol. At 24 h post-transfection, cells were induced to differentiate into adipocytes with adipogenic differentiation medium with 10 μM ATRA or 10 μM SR11237. After 24 h or 48 h, the Luciferase activity assay was performed using the Dual-Luciferase^®^ Reporter (DLR™) Assay System (Promega). Luciferase reporter activity was normalized to Renilla luciferase activity. Transfection efficiency was estimated by co-transfecting with the plasmid-expressing green fluorescent protein. Data are representative of three independent experiments.

### 4.7. Transient Transfection of shRNA

hBMSCs were seeded in six-well plates 24 h before infection and were approximately 50% confluent on the day of infection. On the day of infection, the medium was replaced with complete medium with Polybrene at a final concentration of 10 μg/mL, and RARβ shRNA lentiviral particles (SC-29466-V, Santa Cruz Biotechnology, Santa Cruz, CA, USA), control shRNA lentiviral particles (SC-108080, Santa Cruz, CA, USA), or copGFP control lentiviral particles (SC-108084, Santa Cruz Biotechnology, Santa Cruz, CA, USA) were added to the culture and incubated overnight. The culture medium was removed and replaced with 1 mL of complete medium (without Polybrene) after 24 h. The copGFP control was used to determine the infection efficiency of the virus. Cells were split 1:2 when they were 80% confluent and were then incubated for a further 24–48 h in complete medium. Next, 2 μg/mL of puromycin (Sigma, St. Louis, MO, USA) was added for selection, and the medium was replaced with fresh puromycin-containing medium every three days until resistant colonies could be identified. qPCR and Western blotting were used to determine the efficiency of the *RARB* knockdown.

### 4.8. Statistical Analyses

SPSS 19.0 software (SPSS, Inc., Chicago, IL, USA) was used for the evaluation of significant differences, with significance set at *p* < 0.05. All data are reported as the mean ± standard deviation (SD). Gene expression data were evaluated using independent sample t-tests, and all other data were evaluated by one-way analysis of variance (ANOVA) with least significance difference (LSD) if the variance was homogeneous, or with Dunnett’s T3, if it was not homogeneous.

## 5. Conclusions

**Figure 7:**
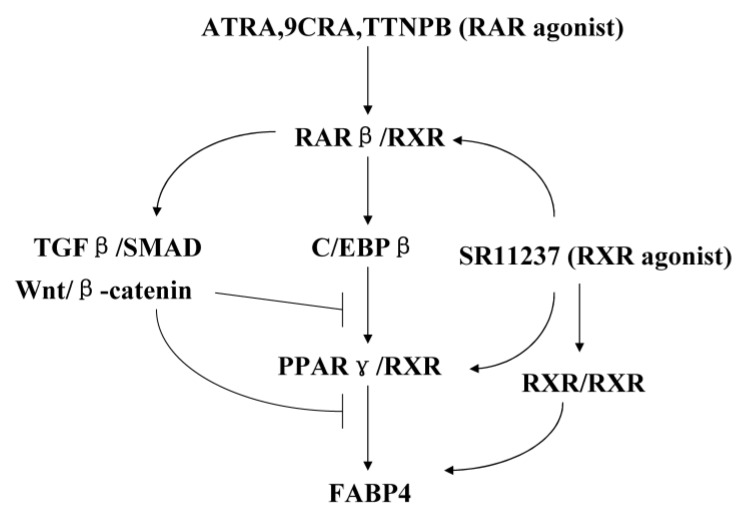
Schematic diagram of a possible mechanism for the regulation of adipogenic differentiation by RAR agonists and RXR agonists in hBMSCs. RAR agonists (ATRA, 9CRA, and TTNPB) stimulate the expression of RARβ, RARβ/RXR heterodimers upregulate the expression of C/EBPβ and promote the adipogenesis, while upregulating the expression of genes related to the TGFβ/SMAD signaling pathway and canonical Wnt pathway, including WNT2B and β-catenin, and block the function of C/EBPβ and PPARγ. RXRs are common heterodimerization partners and RXR agonists’, such as SR11237, can activate RAR/RXR, PPARγ/RXR and RXR/RXR homodimers, final effects in adipogenic differentiation may depend on the relative levels and activities of partners binding to RXRs, including the RARs and PPARγ.

In summary, we evaluated the different effects of RAR agonists and RXR agonists on adipogenesis. We demonstrate, for the first time, that RAR agonists played dual roles in the regulation of adipogenesis in hBMSCs. RAR agonists upregulated the expression of C/EBPβ and promoted adipogenesis in the early stages, but inhibited the adipogenesis in the later stages of adipogenesis. The upregulation of expression of the TGFβ/SMAD pathway and Wnt/β-catenin pathway by RAR agonists may potentially block the function of C/EBPβ and PPARγ. We found that the main RAR subtype mediating this effect is RARβ, although some reports have shown that RAR agonist regulates adipogenesis through RARγ and RARα in some cell systems [8,25]. In contrast, RXR agonists significantly promoted adipogenesis in hBMSCs, but inhibited it in SW872 cells, displaying cell type-specific differences. The final effects of RXR agonists may be based on the integration of signals from different RXR dimers. (Figure 7).

## Figures and Tables

**Table 1:** Sequences of primers for qRT-PCR.

Gene Name	Forward Primer Sequence	Reverse Primer Sequence
*ACTB*	5′ GCGAGAAGATGACCCAGATCATGT 3′	5′ TACCCCTCGTAGATGGGCACA 3′
*ADIPOQ*	5′ CATCTCCTCCTCACTTCCATTCTG 3′	5′ GCCCTTGAGTCGTGGTTTCCT 3′
*FABP4*	5′ GGATGATAAACTGGTGGTGGAATG 3′	5′ CAGAATGTTGTAGAGTTCAATGCGA 3′
*LPL*	5′ AGGAAAGGCACCTGCGGTAT 3′	5′ ACTTTTGAAACACCCCAAACACT 3′
*CEBPB*	5′ CAGGAGAAACTTTAGCGAGTCAGA 3′	5′ GGGTGGCCGCTATTAGTGAG 3′
*CEBPA*	5′ CCAAGAAGTCGGTGGACAAGAAC 3′	5′ CACCTTCTGCTGCGTCTCCA 3′
*PPARG*	5′ GGGATGTCTCATAATGCCATCAG 3′	5′ GCCCTCGCCTTTGCTTTG 3′
*RARA*	5′ GACCTGGTCTTTGCCTTCG 3′	5′ CTCCGCTTCCGCACGTAG 3′
*RARB*	5′ TGGATTGACCCAAACCGAA 3′	5′ GAGGGGGAGGAAGTGGAGAT 3′
*RARG*	5′ GCCATCTGCCTCATCTGCG 3′	5′ CGGAGGTCGGTGATTTTCATTA 3′
*TGFB1*	5′ CCCACAACGAAATCTATGACAAG 3′	5′ GAGGTATCGCCAGGAATTGTTG 3′
*TGFB2*	5′ GCATAAGGAGGCGGGAATC 3′	5′ TGGGTTACATCCAACACAAGG 3′
*TGFB3*	5′ CCCCTGAGCCAACGGTGA 3′	5′ GTTCGTTGTGCTCCGCCA 3′
*SMAD1*	5′ GCCGAATGCCTTAGTGACAG 3′	5′ CTCCCCAGCCCTTCACAA 3′
*SMAD2*	5′ CTGGAGAATAACAGATGGGATGC 3′	5′ CCCTGGCTCCTCACTTGGC 3′
*SMAD3*	5′ GTGCTACATAGGTGCTTTGGGC 3′	5′ GCGGTCGTGTTGACTAGGTGAA 3′
*SMAD4*	5′ CTTCAGGGGCTTCTAAAACAG 3′	5′ TATCAGAGAGGGAAGAGACCAG 3′
*SMAD5*	5′ TGGAGAGGTGTATGCGGAATG 3′	5′ TCCCCAACCCTTGACAAAACT 3′
*WNT1*	5′ CGTCCCGTCCCACCGTC 3′	5′ AGGCAACAGCGCCCAGAG 3′
*WNT2B*	5′ GCTGTGGTCGCACGGCTG 3′	5′ ATAGCGTCGCCGCAGGTAAT 3′
*WNT4*	5′ GCACCATGAGTCCCCGCTC 3′	5′ GATGCTCCCCACCGACGAC 3′
*WNT5A*	5′ TGAACCTGCACAACAACGAG 3′	5′ ATCACCCACCTTGCGGAA 3′
*WNT5B*	5′ CCAAGACTGGCATCAAGGAAT 3′	5′ GTCTCTCGGCTGCCTATCTG 3′
*CTNNB1*	5′ AGGCTACTGTTGGATTGATTCG 3′	5′ CGCTGGGTATCCTGATGTGC 3′
